# Global-Local Spatial-Temporal Residual Correlation Network for Urban Traffic Status Prediction

**DOI:** 10.1155/2022/7344522

**Published:** 2022-02-02

**Authors:** Yin-Xin Bao, Yang Cao, Qin-Qin Shen, Quan Shi

**Affiliations:** ^1^School of Information Science and Technology, Nantong University, Nantong 226019, China; ^2^School of Transportation and Civil Engineering, Nantong University, Nantong 226019, China

## Abstract

The recent proposed Spatial-Temporal Residual Network (ST-ResNet) model is an effective tool to extract both spatial and temporal characteristics and has been successfully applied to urban traffic status prediction. However, the ST-ResNet model only extracts the local spatial characteristics and ignores the very important global spatial characteristics. In this paper, a novel Global-Local Spatial-Temporal Residual Correlation Network (GL-STRCN) model is proposed for urban traffic status prediction to further improve the prediction accuracy of the existing ST-ResNet model. The GL-STRCN model firstly applies Pearson's correlation coefficient method to extract high correlation series. Then, considering both global and local spatial properties, two components consisting of 2D convolution and residual operation are used to capture spatial features. After that, based on Long Short-Term Memory (LSTM) or Gated Recurrent Unit (GRU), a novel long-term temporal feature extraction component is proposed to capture temporal features. Finally, the spatial and temporal features are aggregated together in a weighted way for final prediction. Experiments have also been performed using two datasets from TaxiCD and PEMS-BAY. The results indicated that the proposed model produces a better prediction performance compared with the results based on other baseline solutions, e.g., CNN, ST-ResNet, GL-TCN, and DGLSTNet.

## 1. Introduction

Real-time and accurate traffic prediction is one of the most important aspects in Intelligent Transportation Systems (ITS) [1]; it can provide traffic managers with traffic information in the near future. Knowing reliable traffic information (e.g., flow, velocity, density, and status) in advance can help traffic managers make scientific traffic signal interval, guide travellers to carry out better routing plans, ease traffic congestion, and eventually reduce carbon emissions. Therefore, it is pivotal to have high accuracy traffic prediction model in modern ITS [2, 3].

The performance of traffic prediction models is affected by both internal and external factors. The internal factors are indicated by the spatial-temporal characteristics, and the external factors include uncertain events such as weather, accidents, and festivals. The purpose of traffic prediction is to use historical data and take the above factors into account to predict the traffic status in the near future. However, traffic prediction is a challenging issue in practice, affected by the following specific complex factors:

  Historical data correlation: the future traffic data is predicted by the model based on historical data. How to scientifically select historical data is very important. Generally, if we want to predict the traffic flow on Wednesday, we use data from Monday or Tuesday into the model, and the accuracy of prediction is expected to be higher than using data from the previous weekend. At present, most of the current methods ignore the relevance of historical data and lack the scientific nature of data filtering.
  Global spatial correlation: most of the existing works on spatial feature extraction focus on local features and ignore global features. For example, when a traffic congestion occurs at a cross section, there will be a significant increase of vehicles at the surrounding intersections, but the total number of vehicles on the whole road network is still constant. In this case, the method of extracting only local features will think that the global traffic flow is also increasing, which is inconsistent with the actual situation.
  Long-term temporal correlation: urban traffic data not only have random characteristics in the short term, but also have periodic characteristics in the long term. For example, a traffic congestion at an intersection lasts for only a short period. Observing over a long-time interval, the vehicle volume at the intersection is still in a stable periodic pattern. Some traditional methods use convolution to extract temporal features, which is good for capturing short-term features, but it is easy to lose long-term features.

A large number of traffic prediction methods have been proposed in the last few decades. Based on different prediction intervals, these methods can be divided into long-term and short-term ones. The long-term traffic prediction focuses on the establishment of macroplanning for the development of traffic facilities, and the short-term traffic prediction tries to make estimate traffic data for the next hour [4].

According to different theoretical structures, these methods can be generally divided into two categories [5, 6], i.e., the model-driven methods and the data-driven methods. The former category is based on mathematical theory, which uses a small amount of data samples to determine model parameters. The common model-driven methods include Autoregressive Integrated Moving Average model (ARIMA) [7], Kalman filter model [8], and grey model [9]. However, these methods are generally presented by simplified and solidified model structure involving ideal hypothesis. Therefore, the prediction accuracy of model-driven methods is not high when they are applied in practice. Different from the model-driven methods, the data-driven methods are based on real-time traffic data and use the machine learning technique to process the data [10]. The data-driven methods can be further divided into traditional machine learning methods and deep learning methods [11, 12].

Traditional machine learning methods include Bayesian model [13] and support vector machine (SVM) [14]. The traditional machine learning methods can deal with the traffic routine better in contrast to model-driven methods. However, its effectiveness is limited when it is used to process high-dimensional data [15]. The rise of deep learning theory makes it possible to process high-dimensional traffic data [16]. In recent years, deep learning based traffic prediction methods have been developed rapidly [17, 18]. Li et al. proposed a model based on ensemble empirical mode decomposition and random vector functional link network to predict travel time [19]. In order to avoid the influence of imbalance and lack of large training samples for the model, Lin et al. proposed incident detection framework based on generative adversarial network (GAN) [20]. These methods can effectively deal with high-dimensional traffic data and show higher prediction accuracy for expressway and carriage way [21]. However, unlike highways and carriage roads, urban traffic data has complex spatial-temporal correlation [22]. Due to the simple structure of the models mentioned above, it is difficult to investigate the spatial-temporal characteristics for complex road networks [23].

Effective extraction of spatial-temporal characteristics is essential to improve the performance of traffic prediction models. The past research focused on extracting the spatial features of traffic data; the spatial structure can be divided into Euclidean structure and non-Euclidean structure [24, 25]. Since the structure of the Euclidean urban traffic data is similar to storage structure of images, and the convolutional neural network (CNN) is one of the most popular models for processing image data [26], CNN has been widely applied to extract spatial characteristics of Euclidean urban traffic data. Khajeh et al. [27] considered the spatial relationship between traffic data, used this spatial information to train CNN, and obtained satisfactory prediction results. CNN has good ability of extracting spatial feature when Euclidean data structure is employed, but it cannot directly process non-Euclidean structure data [28]. To extract spatial characteristics from data of non-Euclidean structure, motivated by CNN [29], Graph Neural Network (GNN) [30] and Graph Convolutional Network (GCN) [31] had been proposed to investigate complex spatial topological structure. The methods mentioned above only focused on the spatial feature extraction but neglected the important temporal features. To remedy this, Recurrent Neural Network (RNN) [32] and its two variants, i.e., LSTM [33] and GRU [34], are widely used to capture the temporal characteristics. However, urban traffic data has complex spatial-temporal characteristics because interdependence, CNN, GCN, LSTM, and GRU have not considered the joint influence of spatial-temporal characteristic [35], which is one of the main reasons for its low accuracy.

To explore the influence of spatial-temporal characteristics, a spatial-temporal hybrid model Convolutional LSTM (ConvLSTM) was proposed [36]. He et al. [37] added residual units to solve the problem that effectiveness of prediction deteriorated with the depth of the model network. Zhang et al. [38] proposed the ST-ResNet, which transformed urban traffic situation into raster data of Euclidean structure and improved the model's ability of capturing both the spatial and temporal characteristics. To improve the model's ability of automatically capturing spatial-temporal characteristics, Bao et al. [39] considered the influence of bad weather on traffic flow in the model. Then, Guo and Zhang [40] further considered external factors such as weather and holidays to achieve the prediction accuracy under external disturbances. Experiment results showed that ST-ResNet and derived variations can effectively extract both spatial and temporal characteristics of urban traffic data. However, the original ST-ResNet and its variations can only extract the local spatial features, neglecting the joint influence of the global and local spatial features.

In order to consider the impact of global spatial features on traffic data, Ren et al. [41] proposed global-local temporal convolutional network (GL-TCN) to capture global and local dynamics, but they ignored the analysis of data correlation in their work. Feng et al. [42] proposed Dynamic Global-Local Spatial-Temporal Network (DGLSTNet) to derive the global and local information simultaneously from both spatial and temporal perspectives, but they ignored the capture of long-term temporal features. Therefore, how to improve correlation in traffic data and long-term temporal correlation is very important to improve performance of traffic prediction model.

To overcome the above shortcomings and extract the correlation information in traffic data, global spatial correlation, and long-term temporal correlation of urban traffic data, a novel Global-Local Spatial-Temporal Residual Correlation Network (GL-STRCN) is proposed. Our work in this paper focuses on prediction method on urban traffic status; the main contributions are the following:

- A spatial-temporal correlation feature extraction component is proposed to ensure that the data processed by the model is coherent.
- We design global, local, and temporal feature extraction components to capture spatial-temporal feature of traffic data.
- We design a comfort function to quantitatively measure additional factors such as weather and accidents.
- We use TaxiCD and PEMS-BAY datasets to verify the accuracy of the newly proposed GL-STRCN model. Experimental results show that the prediction performance of our proposed model is the best one when compared with other baseline models, including CNN, ST-ResNet, GL-TCN, and DGLSTNet.

## 2. Problem Description

In this section, we first review the definition of traffic raster data, then discuss the spatial-temporal characteristics of urban traffic data, and finally analyze the impact of global-local spatial characteristics.

### 2.1. Definition of Traffic Raster Data

**Figure 1:**
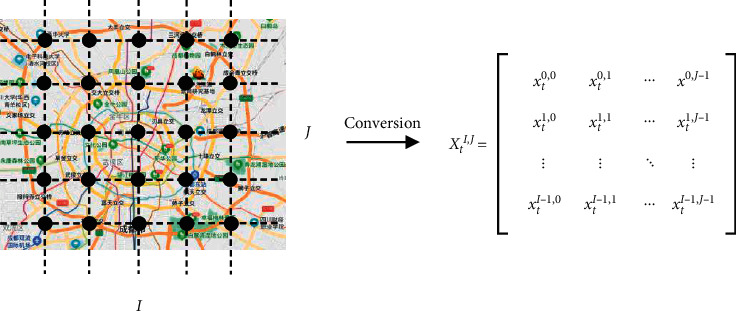
Definition of the raster data.

There are many kinds of spatial-temporal data in our world, Atluri et al. [43] divided spatial-temporal data into four categories, i.e., event data, trajectory data, point reference data, and raster data. Urban traffic data is a typical spatial-temporal data. In this article, we mainly study the traffic data with a raster structure. The definition of raster data is shown in Figure 1.

We firstly transform urban traffic data into an *I∗J* Euclidean structural based on latitude and longitude. Thus, each position in the network is regularly distributed. The relationship between points is similar to that of pixels in an image. Secondly, we record the traffic data of each location in the network at a fixed time interval ∆*t*. *x*_*t*_^*i*,*j*^ represents the urban traffic data collected in the location (*i*, *j*) at time *t*, the urban traffic data of the network area *I∗J* is represented by *X*_*t*_ ∈ ℝ^*I*∗*J*^, and *X*_*t*_ is named as the traffic raster data.

### 2.2. Problem Definition

After conversion in Section 2.1, the traffic prediction problem is transformed into the given historical traffic raster data {*X*_*t*_| *t* = 0,…, *k*} and then they are used to derive the data *X*_*k*+∆*t*_ at a later time interval *k* + ∆*t*, where *k* is the last time node for traffic raster data. Traffic raster data not only has traditional spatial-temporal characteristics, but also has significant global-local spatial characteristics. Accurate learning of these characteristics is essential to improve the prediction accuracy of the model.

### 2.3. Spatial-Temporal Characteristics Analysis

**Figure 2:**
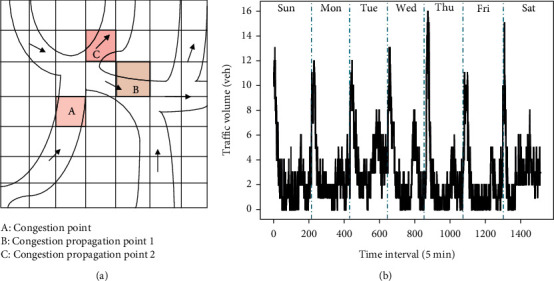
Spatial-temporal characteristics analysis of urban traffic data. (a) Spatial dimension. (b) Temporal dimension.

Urban traffic data is used to generate spatial-temporal characteristics. In the spatial dimension, due to the interconnection between urban road networks, when traffic congestion occurs in a certain area of the road network, the congestion status will be postponed to the surrounding areas, as shown in Figure 2(a). In the temporal dimension, urban traffic data is affected by historical traffic data, and the daily traffic data has some similarity, as shown in Figure 2(b). Therefore, the traffic data at the next moment in a certain area of the urban road is not only related to the traffic data at the previous moment, but also related to the traffic data in the nearby area. Considering a single feature of the urban traffic data only has obvious defects and often results in low prediction accuracy.

### 2.4. Global-Local Spatial Characteristics Analysis of Urban Traffic Data

**Figure 3:**
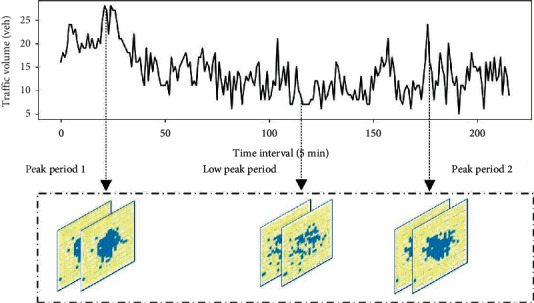
Global spatial characteristics of urban traffic data.

Urban traffic data is affected by both global and local spatial features. From the perspective of the overall traffic status of urban traffic data, during the peak period, the whole urban road network is in a status of congestion, as shown in Figure 3. On the contrary, outside the peak period, the urban traffic status is smooth. Therefore, urban traffic data has significant global spatial characteristics. Urban traffic data also has obvious local spatial characteristics. For instance, if a traffic accident occurs, then the trend of traffic data in its local areas will be greatly changed. Therefore, if we only consider one of the global or local characteristics, the corresponding prediction model may have low accuracy.

## 3. Methodology

In this section, the fundamental architecture of the original ST-ResNet is briefly reviewed first. Then, the framework of proposed GL-STRCN is introduced in detail.

### 3.1. Structure of Classical ST-ResNet

**Figure 4:**
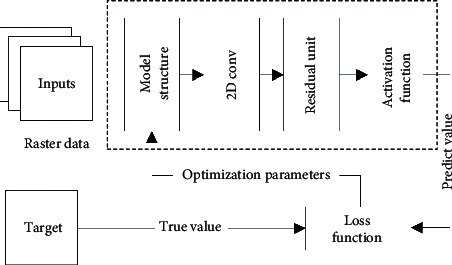
Structure of ST-ResNet.

We introduce the classical ST-ResNet for making the paper self-contained. It is easy to see that the ST-ResNet consists of 2D convolution and residual unit. As discussed in Section 1, ST-ResNet used 2D convolution to extract the spatial characteristics of urban traffic data and combine 2D convolution and residual unit to extract the temporal characteristics; the structure of ST-ResNet is shown in Figure 4.

### 3.2. Global-Local Spatial-Temporal Residual Correlation Network

#### 3.2.1. Basic Structure

**Figure 5:**
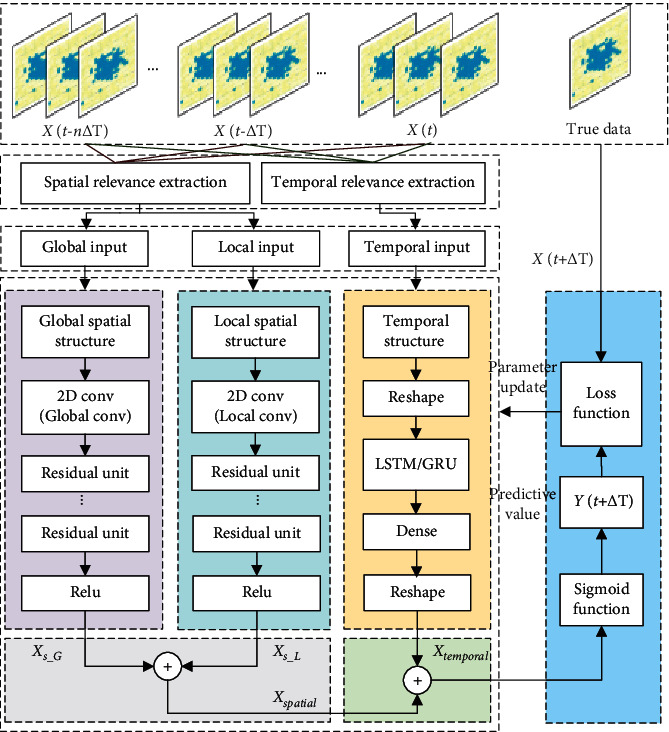
Structure of Global-Local Spatial-Temporal Residual Correlation Network.

As described in Section 2.1, we transform the urban traffic data into traffic raster data and generate traffic raster sequence according to time. Through the establishment of spatial-temporal correlation extraction component, the correlation analysis of historical traffic raster series is carried out, and the series data with high correlation degree is generated into spatial-temporal series. Two kinds of convolution kernels are designed to construct global and local spatial feature extraction components. The global and local spatial features of urban traffic raster data are captured, respectively, and the two features are fused to obtain the spatial feature. Using the temporal feature capture capabilities of LSTM or GRU models, we construct a long-term temporal feature extraction component to obtain the temporal characteristics of traffic raster data. Finally, the spatial and temporal features are weighted out, and the final predicted value is obtained through the activation function. The structure of the GL-STRCN is shown in Figure 5. According to different temporal feature extraction components, two models of GL-STRCN (LSTM) and GL-STRCN (GRU) are obtained.

#### 3.2.2. Spatial-Temporal Correlation Feature Extraction Component

In order to improve the correlation of the input data, Pearson's correlation coefficient method [44] is introduced. Pearson's correlation coefficient formula is

(1) $$\begin{array}{c} \rho_{x,y}=\frac{\left[\sum_{i=1}^{n}\left(x_{i}-\bar{x}\right)\left(y_{i}-\bar{y}\right)\right]}{\left(\sigma_{x}\sigma_{y}\right)}, \end{array}$$

where *x*_*i*_ and *y*_*i*_ (*i* = 1,…, *n*) are the target traffic raster data and the traffic raster data to be compared, respectively, *n* is the number of traffic rasters to be selected, *σ*_*x*_ is the sample population standard deviation of the target traffic raster data, and *σ*_*y*_ is the sample population standard deviation of the traffic raster data to be compared. According to Pearson's correlation coefficient method, the original traffic raster data can be divided into spatial sequence input *X*_inS_ and temporal sequence input *X*_inT_.

#### 3.2.3. Global Spatial Feature Extraction Component

**Figure 6:**
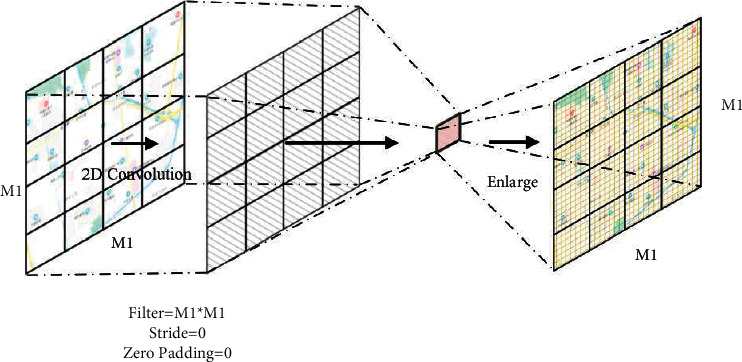
Global spatial feature extraction.

Take the traffic raster data dimension *M*_1_ *∗* *M*_1_; for example, convolution kernel dimension is set to *M*_1_, step is set to 0, and no pooling is done. The global spatial feature convolution operation is shown in Figure 6. The global spatial feature convolution formula is defined as

(2) $$\begin{array}{c} X_{G}^{l}=f_{\text{AF}}\left(f_{\mathrm{EN}}\left(W_{G}^{l}*X_{G}^{l-1}+b_{G}^{l}\right)\right),\;l=1,\ldots ,L_{G}, \end{array}$$

where *X*_*G*_^*l*−1^ and *X*_*G*_^*l*^ are the input and output of the *l*-th layer of the global spatial feature extraction component, respectively, *W*_*G*_^*l*^ is the global convolution kernel, *b*_s_^*l*^ is the bias term of the *l*-th global feature extraction convolutional layer, and *L*_G_ is the number of layers that the global spatial feature extraction component needs to convolute. *f*_EN_ represents a size enlargement operation, enlarging the dimension from 1 *∗* 1 to the dimension of the traffic raster data. *f*_AF_ is the activation function.

**Figure 7:**
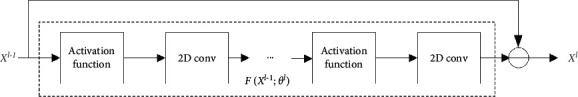
Residual structure.

To avoid the prediction accuracy that decreases as the depth of the convolution layer increases, we introduce residual units to improve the sensitivity of our model to decrease changes in data; the residual operation is shown in Figure 7. The output *X*_*G*_^*l*^ of the global convolution component is input to the residual unit, and the residual operation of the spatial feature extraction component is defined as

(3) $$\begin{array}{c} X_{S}^{l}=X_{S}^{l-1}+F_{R}\left(X_{S}^{l-1};\theta_{S}^{l}\right),\;l=1,\ldots ,L_{R}, \end{array}$$

where *X*_*S*_^*l*−1^ and *X*_*S*_^*l*^ are the input and output of the *l*-th residual unit, respectively, *θ*_*S*_^*l*^ is the set of learnable parameters in the *l*-th residual unit, *F*_R_ is the residual mapping of the global spatial feature extraction component, and *L*_R_ is the number of residual layers required for global components. After the output of the global convolution component is processed by residual operation, the global spatial feature output *X*_sG_ is obtained.

#### 3.2.4. Local Spatial Feature Extraction Component

**Figure 8:**
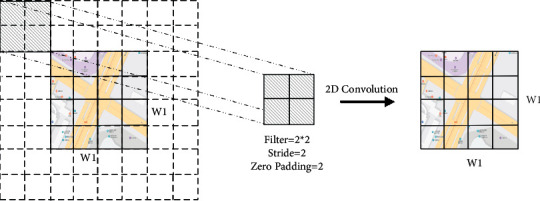
Local spatial feature extraction.

We also construct a local spatial feature extraction component to extract the local spatial characteristics of the traffic raster data. To avoid the insufficient dimensionality, as described in Section 3.2.3, we only convolute the traffic raster data and do not reduce the dimension. We set the size of convolution kernel of local spatial features smaller than the dimension of data to capture local spatial features. The local spatial feature convolution is shown in Figure 8. The local spatial feature convolution formula is defined as

(4) $$\begin{array}{c} X_{\text{L}}^{l}=f_{\text{AF}}\left(W_{\text{L}}^{l}*X_{\text{L}}^{l-1}+b_{\text{L}}^{l}\right),\;l=1,\ldots ,L_{\text{L}}, \end{array}$$

where *X*_*L*_^*l*−1^ and *X*_*L*_^*l*^ are the input and output of the *l*-th layer of the local spatial feature extraction component, respectively, *W*_*L*_^*l*^ is the local convolution kernel, *b*_L_^*l*^ is the bias term of the *l*-th local feature extraction convolutional layer, and *L*_L_ is the number of layers that the local spatial feature extraction component needs to convolute.

Similar to global convolution component, the output *X*_*L*_^*l*^ of the local convolution component is input to the residual unit. After the output of the local convolution component is processed by residual operation, the local spatial feature output *X*_sL_ is obtained.

#### 3.2.5. Long-Term Temporal Feature Extraction Component

Urban traffic data is affected by spatial and temporal characteristics in daily operations. The original ST-ResNet lacks the ability of capturing the long-term characteristics of traffic data, and it is easy to lose the rules of urban traffic data. This paper designs long-term temporal feature extraction components based on LSTM and GRU, respectively, and defines the operation of the time feature extraction component as

(5) Xtem=fRe2fLSTM/GRUfRe1Xtemm,n,

where *X*_tem_^(*m*, *n*)^ is the traffic raster data with dimension (*m*, *n*), *f*_Re1_ is a matrix change operation that changes the dimension of the matrix from (*m*, *n*) to (1, *m* *∗* *n*), *f*_LSTM_ is the forward calculation of LSTM, *f*_GRU_ is the forward calculation of GRU, *f*_Re2_ is a matrix change operation that changes the matrix dimension from (1, *m* *∗* *n*) to (*m*, *n*), and *X*_tem_ is the final output of the temporal feature extraction component.

#### 3.2.6. Fusion of Spatial-Temporal Characteristics

We adopt a parameter matrix fusion method to perform weighted fusion of the global spatial feature output *X*_sG_, local spatial feature output *X*_sL_, and long-term temporal feature output *X*_tem_. The weight value is dynamically adjusted according to model training. The formula is

(6) $$\begin{array}{c} X_{\mathrm{Fusion}}=f\left(W_{\mathrm{sG}}*X_{\mathrm{sG}}+W_{\mathrm{sL}}*X_{\mathrm{sL}}+W_{\mathrm{tem}}*X_{\mathrm{tem}}\right), \end{array}$$

where *W*_sG_, *W*_sL_, and *W*_tem_ represent the proportions of global spatial features, local spatial features, and long-term temporal features, respectively. *f* is a sigmoid function.

#### 3.2.7. Loss Function

The index mean square error (MSE) is used as the loss function to evaluate the errors between the real values and predicted values in model training

(7) $$\begin{array}{c} L_{\mathrm{MSE}}=\frac{\sum_{i=1}^{n}{\left|X_{T}-X_{P}\right|}^{2}}{n}, \end{array}$$

where *X*_T_ and *X*_P_ are the real value and predicted value, respectively, and *n* is the total number of samples.

## 4. Experiments

In order to evaluate the effectiveness of the proposed model, a series of experiments have been conducted. They are organized into the following steps.

### 4.1. Data Collection

#### 4.1.1. TaxiCD

The experimental data records the positioning data of Chengdu taxis from 6:00 a.m. to 12:00 p.m. every day. The specific date is from August 3, 2014 to August 23, 2014, totally 21 days. The data format is shown in Table 1. We use TaxiCD data to verify the prediction accuracy of Euclidean structure models.

#### 4.1.2. PEMS-BAY

PEMS-BAY is the traffic data collected by the performance measurement system of California transportation department. There are totally 325 sensors, which collect traffic data for five months (January 1, 2017 ∼ May 31, 2017). The time interval of data is 5 min. PEMS-BAY is mainly used to verify the prediction accuracy applicable to non-Euclidean structural models.

### 4.2. Construction of Traffic Raster Data

Based on the distribution of vehicles, the original data is converted into traffic raster data by latitude and longitude. Generate traffic raster data at 5-minute sampling intervals. The traffic raster structure of the two datasets is shown in Table 2. To determine whether the latitude and longitude of the vehicle are within the raster range, the discriminant function for mapping the original data to the traffic raster network is designed as follows:

(8) $$\begin{array}{c} f\left(D_{\text{lon},\text{lat}}\left(n\right),x_{i,j}\right)=\left\{\begin{array}{c} \text{Min}\left(\text{lon}\left(x_{i,j}\right)\right)<D_{\text{lon}\left(n\right)}<\text{Max}\left(\text{lon}\left(x_{i,j}\right)\right)\;i\in I,j\in J\\ \text{Min}\left(\text{lat}\left(x_{i,j}\right)\right)<D_{\text{lat}\left(n\right)}<\text{Max}\left(\text{lat}\left(x_{i,j}\right)\right)\;i\in I,j\in J \end{array}\right., \end{array}$$

where Min (lon (*x*_*i,j*_)) and Max (lon (*x*_*i,j*_)) represent the minimum and maximum longitude of the location of the traffic raster network *x*_*i,j*_, respectively, and *D*_lon_ (*n*) represents the longitude of the original data. The notations in the second line of the above formula have the same meanings for the latitude.

After the traffic raster data is generated, we need to standardize the traffic raster data to reduce the influence of different dimensions between the data, and the calculation formula is as follows:

(9) $$\begin{array}{c} X_{\mathrm{nor}}^{n}=\frac{\left(X_{\mathrm{real}}^{n}-{\bar{X}}_{\mathrm{real}}\right)}{\sigma_{x}}, \end{array}$$

where *X*_real_^*n*^ is the *n*-th data in the traffic raster data, ${\bar{X}}_{\mathrm{real}}$ is the average value of all traffic raster data, and *σ*_*x*_ is the standard deviation of the overall traffic raster data.

### 4.3. Extraction of Spatial-Temporal Correlation Sequence

**Figure 9:**
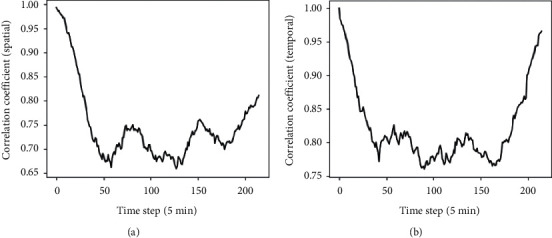
Comparison of temporal and spatial correlation. (a) Spatial correlation. (b) Temporal correlation.

After the generation of traffic raster data, we take the raster data *x*_0,17_ of TaxiCD as an example and use spatial-temporal correlation feature extraction component to analyze its correlation. The correlation curve is shown in Figure 9, and the time step in Figure 9 is five minutes. Figures 9(a) and 9(b) show the spatial and temporal correlation of traffic data over a day, respectively. As shown in Figure 9, the smaller the time interval to the time node to be predicted, the higher the spatial-temporal correlation between the traffic raster data.

### 4.4. Model Parameter Settings

The new proposed GL-STRCN is built based on the deep learning framework PyTorch, and the experiment is carried out on a computer equipped with GPU computing. The Adam optimizer is used to optimize the model parameters. The training step is set to 0.0001, the number of batches is set to 20, and the maximum number of iterations is set to 800; the convolution kernels and residual cells are initialized by random functions. Other structure parameters of the model are shown in Table 3.

## 5. Results

The experiments have been performed based on the steps outlined in Section 4. The performance of the proposed GL-STRCN model is compared with that of four baseline models, e.g., CNN [26], ST-ResNet [38], GL-TCN [41], and DGLSTNet [42]. In particular, the global-local features of the collected data have been analyzed separately. The possible network topologies to be employed in the proposed model and its impact on the results are also investigated. In addition, the reliability of the results is analyzed by considering some external factors.

### 5.1. The Global-Local Predictions Based on the GL-STRCN

**Figure 10:**
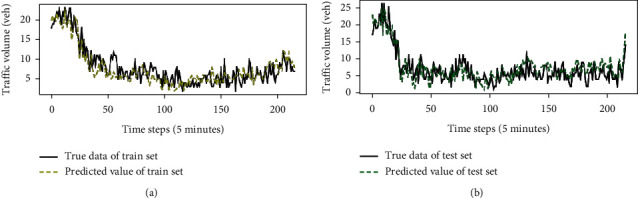
Local predictive effect of GL-STRCN. (a) Predictive effect of model on training set. (b) Predictive effect of model on test set.

**Figure 11:**
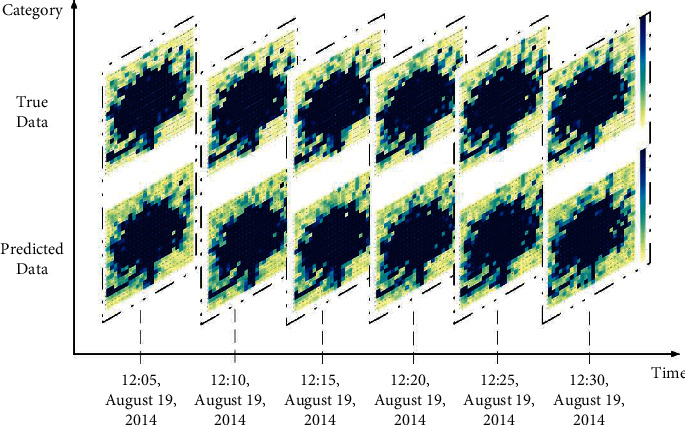
Global predictive effect of GL-STRCN.

The abovementioned GL-STRCN was used as the initial instrument to make predictions. After the initialization process was completed, the parameters of the model are trained with a training set. We take *x*_10,10_ of TaxiCD in traffic raster data as an example. Figures 10(a) and 10(b) show the local prediction effect of the GL-STRCN in the test set and the training set, respectively.  Figure 11 shows the global prediction effect of the GL-STRCN in the test set. In both scenarios, it is observed that the traffic volume on 19 August, 2014 starts from a peak and gradually flattens at the later hours of the day. There is a minor fluctuation at the mid-day time. At the final hours of the day, the fluctuation is becoming stronger. Note that there is no marked discrepancy in raster data found from 12:00 to 12:30 on 19 August, 2014.

### 5.2. Evaluation by Comparing the Results from Two Classical Baseline Models

We choose the CNN and the original ST-ResNet as the template baseline models to evaluate the results from the proposed model. Root Mean Square Error (RMSE) and Mean Absolute Error (MAE) are used to evaluate the prediction performance of above models.

(10) $$\begin{array}{c} E_{\mathrm{RMSE}}=\sqrt{\frac{1}{m}\overset{m}{\underset{i=1}{\sum }}{\left(y_{i}-{\bar{y}}_{i}\right)}^{2}},\\ E_{\mathrm{MAE}}=\frac{1}{m}\overset{m}{\underset{i=1}{\sum }}\left|y_{i}-{\bar{y}}_{i}\right|, \end{array}$$

where *y*_*i*_ is the true value of traffic data, ${\bar{y}}_{i}$ is the traffic data predicted by the model, and *m* is the number of samples.

**Figure 12:**
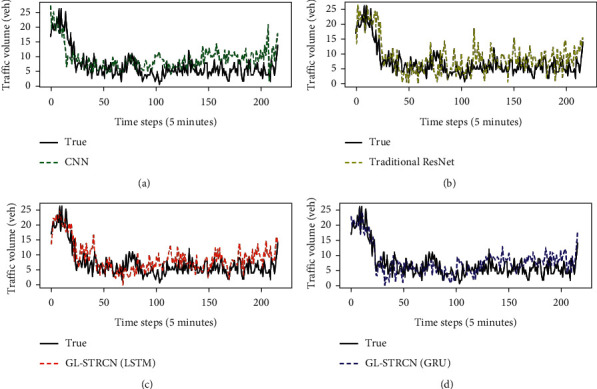
The prediction performance of the four models. (a) CNN. (b) ResNet. (c) GL-STRCN (LSTM). (d) GL-STRCN (GRU).

The baseline models were trained and tested by using the data from TaxiCD, and the parameter settings for each baseline model are the same as those for GL-STRCN (GRU). The prediction results of different models in the test set are shown in Table 4.  Figure 12 shows the results of different models for predicting traffic data on a random day in a test set. It can be seen from Table 4 and Figure 12 that the prediction results of GL-STRCN model are more convergent than CNN and ST-ResNet.

**Figure 13:**
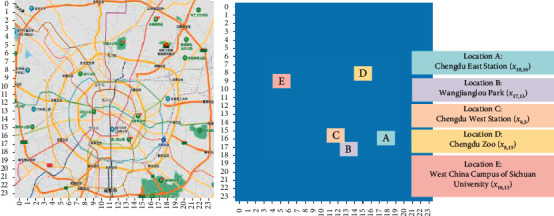
Five typical local regions are used to verify the local feature extraction capabilities of GL-STRCN.

In order to measure the prediction accuracy of the model in greater detail at a local domain, we investigated five small areas in the raster data; they are location A: Chengdu East Station (*x*_18,16_), location B: Wangjiang Tower Park (*x*_17,13_), location C: Chengdu West Railway Station (*x*_9,5_), location D: Chengdu Zoo (*x*_8,15_), and location E: West China Campus of Sichuan University (*x*_16,12_). The distribution of the specific coordinate points is illustrated in Figure 13.

The RMSE values of different prediction models are listed in Table 5. In Table 6, we list the MAE values. Based on the experimental results in Tables 5 and 6, GL-STRCN has the best predictive effect, and the best value is shown in bold. Compared with other baseline models, our model has better accuracy in predicting local traffic status.

**Figure 14:**
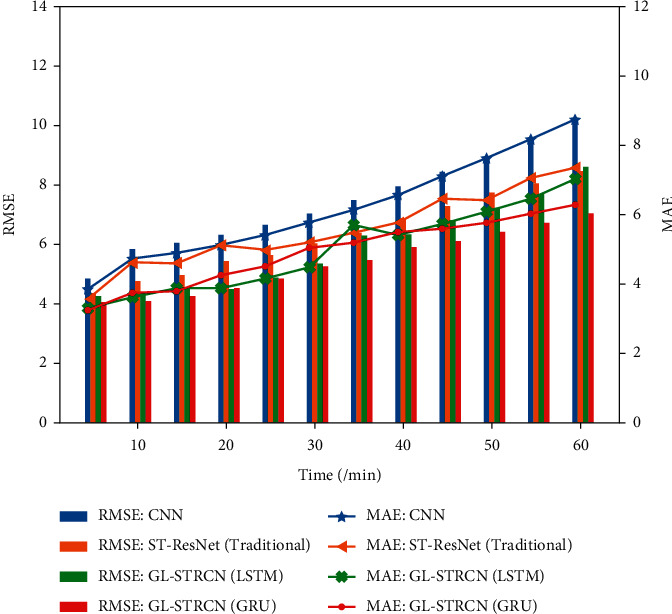
The prediction interval was extended from 5 minutes to 1 hour to verify the long-term prediction ability of GL-STRCN.

To validate the effect of time intervals on model predictions, we increase the prediction interval from 5 minutes to 1 hour to assess the long-term predictive performance of GL-STRCN.  Figure 14 shows the traffic data prediction results for location A. From the graph, the prediction accuracy of all models decreases with the increase of the prediction interval. As the prediction interval increases, the GL-STRCN proposed by the authors always maintains a good prediction accuracy.

### 5.3. Analysis of the Model considering Global-Local Features

**Figure 15:**
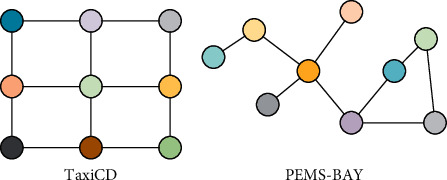
The structures of TaxiCD and PEMS-BAY.

In order to verify the superiority of GL-STRCN in global-local spatial feature extraction, we select GL-TCN and DGLSTNet as baseline models for comparison. The parameter settings of all models are basically the same. We use data of TaxiCD and PEMS-BAY to train and test all models. The structures of TaxiCD and PEMS-BAY are shown in Figure 15.

The prediction results of the discussed three models are shown in Table 7. We see that, for Euclidean and non-Euclidean traffic data, GL-STRCN (GRU) and DGLSTNet have the best prediction accuracy, respectively.

### 5.4. Network Configuration of the GL-STRCN

**Figure 16:**
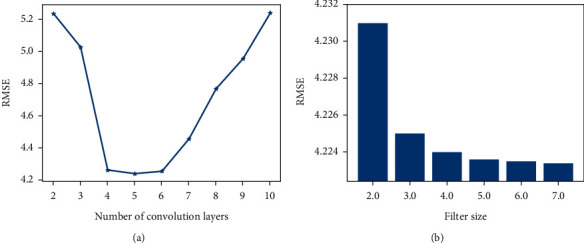
Effect of different network configuration.

In order to verify the influence of the number of convolution layers on the GL-STRCN model, we increase the number of convolution layers from 2 to 10. It can be seen from Figure 16(a) that when the number of convolution layers is 5, the RMSE error is the smallest; when the convolution layers is set greater than 5, the accuracy of the proposed model decreases gradually. By changing the size of the convolution kernel, as shown in Figure 16(b), we find that when the size of the convolution kernel is between 3 and 7, the RMSE accuracy of the model has little difference.

### 5.5. Uncertainty due to the External Factors

**Figure 17:**
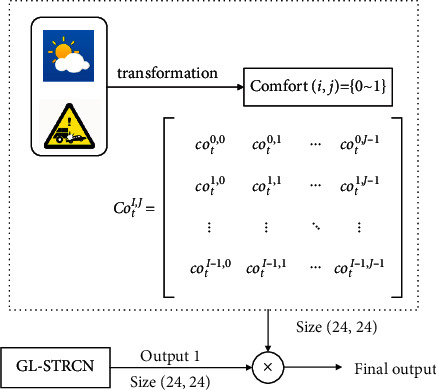
The structure of external interference module.

The daily traffic conditions are complex and unstable. In order to improve the adaptability of the model, we expand the components of GL-STRCN and introduce the external interference module. The structure of external interference module is shown in Figure 17.

In this section, we define a comfort function *f*_comfort_ ∈ [0,1] . When the current weather or traffic condition is satisfactory, *f*_comfort_=1; otherwise it is 0. We use TaxiCD to verify the accuracy of the improved model and use the crawler written in Python to crawl the corresponding weather data of Chengdu. The definition of comfort corresponding to weather is shown in Table 8.

After the weather data processing is completed, it is transformed into a satisfaction matrix with dimensions of (24, 24), and the training accuracy comparison of the test set model is obtained, as shown in Table 9. The model considering additional factors has higher accuracy.

### 5.6. Discussion

From the numerical results shown in Sections 5.1–5.5, we see that our proposed GL-STRCN model shows a significant improvement on the prediction accuracy. In particular, when extracting both global and local features from traffic data, the GL-STRCN (GRU) model shows excellent performance. CNN, as a well-known prediction model, is difficult to effectively extract the temporal characteristics of urban traffic data.

When processing spatial-temporal raster data, the original ST-ResNet lost the long-term temporal characteristics of traffic data due to failing to capture the time trends. LSTM and GRU, as improved models of RNN, effectively solve the problem of gradient disappearance and gradient explosion in RNN. However, the GRU structure is simpler and easier to train than LSTM, which can reduce redundancy and improve training efficiency of the model.

Due to lack of the ability of analyzing historical data association and capturing long-term time characteristics by using the GL-TCN and the DGLSTNet, the prediction accuracy of the above two models in urban road environment datasets is not as good as that of GL-STRCN.

It is noted that choosing the appropriate network parameters is critical to the prediction performance of GL-STRCN. If the interference of external factors is ignored, the prediction performance of the model will also be reduced.

In summary, through a number of experimental comparisons, it is found that the GLSTRCN model proposed in this paper has better prediction performance in urban environment. Compared with other baseline models, GL-STRCN not only effectively extracts global-local spatial-temporal features, but also has the ability of extracting long-term temporal features. Therefore, the GL-STRCN model proposed in this paper is more suitable for urban road network traffic prediction.

## 6. Conclusion

In this paper, we investigated the methods for traffic flow status prediction and proposed a Global-Local Spatial-Temporal Residual Correlation Network (GL-STRCN). Spatial-temporal correlation feature extraction component was built to implement historical data correlation. Global and local spatial feature extraction component was constructed to capture spatial association. Long-term temporal feature extraction component was constructed by using the strong time feature capture capabilities of LSTM or GRU to acquire dynamic time evolution. Two traffic datasets are adopted to verify the prediction accuracy of the proposed GL-STRCN model. Experimental results demonstrated the effectiveness of the new proposed model over the existing methods, in particular in an urban environment. The future work will focus on capturing the spatial-temporal correlation of models in complex traffic environments to improve the accuracy of traffic prediction.

## Figures and Tables

**Table 1:** Original data format of TaxiCD.

Label	Explanation
Taxi_ID	The number of taxis
Lon	The longitude of taxi
Lat	The latitude of taxi
Up_down	Get on or off
Time	Record time

**Table 2:** Format of traffic raster data.

Type	TaxiCD	PEMS-BAY
Location	In Chengdu, China	In California, USA
Date	August 3, 2014, to August 23, 2014	January 1, 2017, to May 31, 2017
Time interval	5 minutes	5 minutes
Raster size	24 *∗* 24	24 *∗* 24
Number of available time intervals	4536	43200
Area of the raster	648 square kilometres	354 square kilometres
Longitude (min)	103.945689	−122.078275
Longitude (max)	104.204976	−121.805543
Latitude (min)	30.585958	37.249226
Latitude (max)	30.786707	37.416413

**Table 3:** Model structure parameters.

Parameter	GL-STRCN
Input size	[Batch size, 1, 24, 24]
Number of residual units	8
Convolution kernel size of global components	24 × 24
Convolution kernel size of local components	3 × 3
Convolution kernel step size of global components	0
Convolution kernel step size of local components	1
Dimensions of the LSTM/GRU input layer	1 × 576
Number of hidden layers of LSTM/GRU	12
Dimensions of the LSTM/GRU output layer	1 × 576
Activation function	Residual unit: ReLu; other: sigmoid

**Table 4:** Comparison of prediction results of different models in the test set (global).

Model	TaxiCD (test set)	
	RMSE (average)	MAE (average)
CNN	4.8555	3.8283
ST-ResNet	4.3478	3.5459
GL-STRCN (LSTM)	4.2578	3.3197
GL-STRCN (GRU)	**4.0295**	**3.2349**

**Table 5:** RMSE results of different prediction models.

Location	Model			
	CNN	ST-ResNet	GL-STRCN (LSTM)	GL-STRCN (GRU)
A	5.7329	4.4519	3.7151	**3.6552**
B	13.4603	13.7236	**6.1164**	6.8788
C	6.0828	5.2430	4.6727	**4.3122**
D	8.9210	4.9526	4.6857	**4.5555**
E	19.4317	15.9623	**5.5641**	6.3349

**Table 6:** MAE results of different prediction models.

Location	Model			
	CNN	ST-ResNet	GL-STRCN (LSTM)	GL-STRCN (GRU)
A	4.6274	4.3776	3.6265	**3.5050**
B	10.6201	13.4834	**6.0850**	6.8516
C	4.8593	5.1226	4.5506	**4.1562**
D	6.3986	4.8172	4.5520	**4.3585**
E	14.9280	15.6158	**5.5062**	6.2832

**Table 7:** Comparison with the model considering global-local features.

Model	TaxiCD	PEMS-BAY
	RMSE (average)	RMSE (average)
GL-TCN	4.0955	2.7335
DGLSTNet	4.1032	**2.6956**
GL-STRCN (GRU)	**4.0295**	2.7224

**Table 8:** Comparison of model accuracy after considering external factors.

Weather	Comfort
Sunshine fine	1
Cloudy	0.8
Overcast sky	0.7
Sprinkle	0.5
Middle rain	0.4
Drencher	0.3
Cyclone	0.1

**Table 9:** Comparison with the model considering external factors.

Model	RMSE in TaxiCD
GL-STRCN (no external factors)	4.1437
GL-STRCN (external factors)	4.0233

## Data Availability

The data used to support the findings of this study are available from the last author upon request.
